# Topical Anesthesia for Cataract Surgery: The Patients' Perspective

**DOI:** 10.1155/2014/827659

**Published:** 2014-06-24

**Authors:** Aytekin Apil, Baki Kartal, Metin Ekinci, Halil Huseyin Cagatay, Sadullah Keles, Erdinc Ceylan, Ozgur Cakici

**Affiliations:** ^1^Department of Ophthalmology, Bakirkoy Dr. Sadi Konuk Training and Research Hospital, Istanbul, Turkey; ^2^Department of Ophthalmology, Regional Training and Research Hospital, Erzurum, Turkey; ^3^Department of Ophthalmology, Medical Faculty of Kafkas University, Kars, Turkey; ^4^Department of Ophthalmology, Medical Faculty of Ataturk University, Erzurum, Turkey; ^5^Department of Ophthalmology, Medical Faculty of Medeniyet University, Istanbul, Turkey

## Abstract

*Purpose*. To evaluate the analgesic efficacy of 0.5% propacaine hydrochloride as topical anesthesia during phacoemulsification surgery. * Methods*. Intraoperative pain intensity was assessed using a 5-category verbal rating scale during each of three surgical stages. Pain scores from each surgical stage and total pain scores were compared for the factors of patient age, gender, cataract laterality, and type. *Results*. In comparison of cataract type subgroups, the mean total pain scores and mean stage 2 pain scores in both white mature cataract (WMC) and corticonuclear plus posterior subcapsular cataract (CN + PSC) groups were significantly higher than in the PSC-only (PSC) group (*P* < 0.05). *Conclusion*. Phacoemulsification with topical anesthesia is not a completely painless procedure. Pain intensity varies with cataract type and stage of surgery.

## 1. Introduction

For routine cataract surgery, topical anesthesia is preferred because it provides sufficient patient comfort with lower incidence of complications compared to other types of anesthesia [1, 2].

The three most common methods of applying topical anesthesia are by eye drops, by eye drops with intracameral lidocaine injection, and in gel form [3, 4]. Topical anesthesia by eye drops is a noninvasive method, but in some cases it may provide insufficient analgesia and require an additional intracameral lidocaine injection [5].

This study aimed to determine the efficacy of topical anesthesia by 0.5% propacaine hydrochloride in controlling pain and providing intraoperative comfort for patients undergoing phacoemulsification.

## 2. Materials and Methods

This prospective study included 63 eyes from 63 patients who presented at the Erzurum Regional Training and Research Hospital between March 1st, 2011, and October 1st, 2012. These patients had no medical history of ocular surgeries or pathologies such as glaucoma, traumatic cataract, or high myopia.

The pain scoring system was based on the Keele verbal pain chart [6] (Table 1). Each patient was informed about the pain scoring system before surgery and was asked to use the scoring system to describe their pain levels during surgery.

Patients were grouped according to age, gender, laterality, and cataract type. The pain score for each surgical stage and total pain score were compared between groups. There were three age groups: 40–59, 60–75, and 76–97 years old. The three categories of cataract were white mature cataract (WMC), posterior subcapsular cataract (PSC), and corticonuclear plus posterior subcapsular cataract (CN + PSC).

The American Optometric Association's grading system for cataracts [7] was used to identify cataract types PSC and CN + PSC. Patients in these groups had stage 2 or 3 cataracts of their respective type according to the AOA's grading system. Criteria for inclusion in the WMC group were total opacity and whiteness of the lens and inability to distinguish epinucleus from nucleus preoperatively or intraoperatively. Severely emulsified epinuclear component or hypermature or morgagnian cataract was not detected preoperatively or intraoperatively in any patients in this group. Also, these patients had no lens to iris contact and their pupil movements were normal in preoperative examinations.

None of the patients received sedation prior to surgery, and each patient underwent the same three-stage procedure performed by a single surgeon (Table 2). Patients spontaneously reported their intraoperative pain levels; these pain scores and the corresponding surgical stages were recorded by surgeons observing the procedure by live video. If patients reported more than one pain score during any surgical stage, the highest value was used as the pain score for that stage. The total pain score is the sum of the pain scores from the three surgical stages.

During the procedure, care was taken to avoid conjunctival manipulation.

Immediately after the operation, the eye was closed and the patient was moved to the inpatient clinic. In the clinic, patients were interviewed using the questionnaire below. They were asked to rate the success of their procedure and explain why they answered as they did.

How do you think your surgery went? Why do you think this way?

I think my surgery went well, because...
I don't think my surgery went well, because...
(c) I don't have an opinion about whether my surgery went well or not.

The pain score data were analyzed statistically using SPSS 17.0 software. The variables were compared using Kruskal-Wallis, Mann-Whitney, and chi-square tests. Differences were considered significant at *P* < 0.05.

The study was approved by the Regional Committee for Medical Research Ethics of Erzurum Training and Research Hospital, Turkey.

## 3. Results

The 63 patients had an average age of 69.27 ± 12.91 years (range: 40–97 years). The age distribution of the patients was as follows: 17 patients between 40 and 59 years; 25 patients between 60 and 75 years; 21 patients between 76 and 97 years. There were 32 men (50.7%) and 31 women (49.2%). The cataract type distribution was as follows: WMC, *n* = 21; PSC, *n* = 20; CN + PSC, *n* = 22. The laterality distribution was 28 right eyes (44.4%) and 35 left eyes (55.5%). The procedures were performed without any complications.

During surgery, 56 patients (88.9%) received only topical anesthetic drops, whereas 7 patients (11.1%) described severe or unbearable pain and received additional intracameral lidocaine injections (Table 3). Of the patients who received lidocaine injection, three had WMCs, one had PSC, and three had CN + PSCs. The pain scores of these patients were not included in the within-group statistics and were analyzed separately. For all patients who received intracameral lidocaine injection, their pain was completely relieved within the first 10 seconds, and they experienced no further intraoperative pain.

When the pain scores from all surgical stages of all 63 patients were analyzed, 6 patients (10.5%) experienced no pain throughout the entire procedure (PSC, *n* = 3; CN + PSC, *n* = 3). The analysis revealed that all patients in the WMC group experienced pain in one or more stages of the surgery.

In patients who received only topical anesthesia, the average total pain score was 3.05 ± 1.24 (0–5); the average for stage 1 was 0.75 ± 0.43 (0-1), for stage 2 was 1.27 ± 0.67 (0–2), and for stage 3 was 1.04 ± 0.5 (0–2). Kruskal-Wallis nonparametric test was used to analyze the relation between cataract type and both mean total pain score and mean pain score per stage (statistics, Tables 4, 5(a), and 5(b)). The mean total pain scores and mean stage 2 pain scores of WMC and CN + PSC groups were significantly higher compared to those in the PSC group when analyzed by Kruskal-Wallis and Mann-Whitney nonparametric tests (*P* < 0.05).

The mean total pain scores and mean pain scores from each surgical stage showed no significant differences between age groups when analyzed by Kruskal-Wallis nonparametric test (*P* > 0.05) (Tables 6(a) and 6(b)).

The mean total pain scores and mean pain scores from each surgical stage showed no significant differences between gender groups and between laterality groups when analyzed by chi-square test (*P* > 0.05) (Tables 7, and 8).

Among all cataract types, there were a total of six patients (9.5%) who felt no pain during the procedure, three in each of the PSC and CN + PSC groups; all of the patients in the WMC group experienced some level of pain.

Of the 63 patients who completed the postoperative questionnaire, 48 patients (76.1%) believed that their procedure had been successful, 5 patients (7.9%) believed that it had been unsuccessful, and 10 patients (15.8%) had no opinion. Of the 48 patients who considered their procedure successful, 33 patients (75%) gave different explanations for their opinion, but 15 patients (23.8%) gave similar answers. These 15 patients belonged to the WMC group, and it became apparent that their perception of surgical success was based on the fact that they experienced an immediate visual improvement when the white mature cataract was removed. All five patients who believed that their procedure was unsuccessful had received a lidocaine injection; the reason that they felt their surgery had been unsuccessful was based on the pain that they experienced during the procedure (three in the CN + PSC group and one in each of the PSC and WMC groups).

## 4. Discussion

Clear corneal phacoemulsification surgery has been the subject of many studies [8–11]. The advantages of topical anesthesia are early recovery of sight and lack of injection-related complications seen with peribulbar or retrobulbar anesthesia [12–14].

In this prospective randomized study, we evaluated the effects of cataract type, age, gender, and laterality on the efficacy of topical 0.5% propacaine hydrochloride anesthesia in providing patient comfort during phacoemulsification.

Soliman et al. reported that 73.3% of patients that received topical 0.4% benoxinate and 10% of patients that received topical 0.5% bupivacaine during phacoemulsification surgery had severe to unbearable pain which led to addition of subtenon lidocaine injection [4]. In our study, seven patients (14.2%) experienced severe to unbearable pain which necessitated intracameral lidocaine injection. In all cases, the severe to unbearable pain occurred in stage 2 of the procedure.

Analysis of the data from 56 patients who received only topical anesthetic drops revealed that the mean total pain scores and mean stage 2 pain scores in both WMC and CN + PSC groups were significantly higher than in the PSC group (*P* < 0.05). This was thought to be referred pain caused by mechanical effects of nucleus rotation or intracapsular manipulation on surrounding tissue, especially the corpus ciliare region, which were necessary due to the high density of the cataracts.

In a study by Malecaze et al. the efficacy of intracameral mepivacaine as a supplement to topical anesthesia during phacoemulsification was investigated. They reported that, within 10 seconds after the intracameral injection, the pain scores of 84% of the patients decreased by at least one level on the Keele verbal score. From this group of patients, 90.4% continued to have decreased pain sensation for the remainder of the procedure, while 9.6% required additional intracameral mepivacaine injection due to increasing pain [15]. In our study, intracameral lidocaine injection resulted in complete pain relief within 10 seconds, and the patients reported no further pain during the remainder of the procedure.

In a study by Kaluzny et al., the analgesic efficacy of oral acetaminophen as a supplement to topical anesthetic drops (0.5% tetracaine) during phacoemulsification was investigated. They reported that the mean verbal pain score of 80 patients in the oral placebo group was 1.11 ± 0.73 [16]. In our study, the mean pain score of 56 patients who only received topical anesthesia was 3.05 ± 1.24 (0–5). The reason for this large difference is that the highest reported score from each of the three stages was added to calculate the total pain score for each patient in our study. If the highest pain score throughout the entire procedure is taken as the pain score of that patient, as in the study by Kaluzny et al., the mean pain score in our study decreases to 1.01 ± 0.41.

The analysis of the questionnaire showed that 15 patients considered their procedure successful because their visual clarity improved during surgery upon cataract removal. It is noteworthy to mention that all of these patients were from the WMC group. WMC blocks more light compared to other types of cataract; therefore, phacofragmentation of the cataract during surgery significantly changes the patients' perception of the brightness of the microscope lamp. This change may have led the patients to conclude that their surgery was successful. Another point of note is that the five patients that required additional lidocaine injection all considered their procedure unsuccessful due to feeling severe or unbearable pain during their surgery.

In conclusion, phacoemulsification with topical anesthetic eye drops is not a completely painless procedure. The majority of patients feel mild or moderate pain, and patients with dense cataracts are more likely to experience severe to unbearable levels of pain. Our data suggest that intense pain leads patients to believe that their procedure was unsuccessful, whereas immediate visual improvements during surgery lead to a belief that the procedure was successful. Therefore, patients need to be informed preoperatively that their visual clarity or pain sensations do not reflect the success of the procedure.

## Figures and Tables

**Table 1:** Pain intensity scoring system.

Intensity	Description	Score
None		0
Mild	Momentary mild sensations of burning or piercing	1
Moderate	Intermittent moderate sensations of burning, piercing, or fullness/tightness in the eye lasting a few seconds	2
Severe	Continuous sensations of piercing or swelling/stretching in the eye severe enough to require additional intervention	3
Unbearable	Continuous sensations of piercing or swelling/stretching of the eye severe enough to make the patient want to stop the procedure	4

**Table 2:** Surgical stages.

Stage 1	Topical anesthesia (0.5% propacaine) application, side port incision, air/dye injection, viscoelastic injection, preincision and clear corneal tunnel incision, and capsulorhexis
Stage 2	Hydrodissection, phacoemulsification by divide-and-conquer method, and corneal rinsing by coaxial irrigation/aspiration
Stage 3	Filling with viscoelastic, one-piece hydrophilic acrylic IOL in-the-bag implantation through insertion tube, viscoelastic removal by irrigation/aspiration, and stromal hydration

**Table 3:** Cataract type, surgical stage, and pain scores in patients requiring supplemental lidocaine injection.

Cataract type	Surgical stage	Pain score	Number of patients
PSC	Stage 2, during nucleus fragmentation and rotation	4	1
CN + PSC	Stage 2, during nucleus fragmentation and rotation	3-4	3
WMC	Stage 2, during nucleus fragmentation and rotation	3	3

**Table 4:** Descriptive statistics.

	N	Mean	Std. deviation	Minimum	Maximum
Total pain score	56	3,05	1,242	0	5
Stage 1	56	,75	,437	0	1
Stage 2	56	1,27	,674	0	2
Stage 3	56	1,04	,503	0	2

Std. deviation: standard deviation, *N*: number.

**(a):** 

Ranks			
	Cataract type	*N*	Mean rank
Total pain score	PSC	19	20,16
	WMC	18	32,92
	CN + PSC	19	32,66
	Total	**56**	

Stage 1	PSC	19	26,66
	WMC	18	29,28
	CN + PSC	19	29,61
	Total	**56**	

Stage 2	PSC	19	19,11
	WMC	18	34,61
	CN + PSC	19	32,11
	Total	**56**	

Stage 3	PSC	19	26,34
	WMC	18	30,33
	CN + PSC	19	28,92
	Total	**56**	

*N*: number.

**(b):** 

Statistics^a,b^				
	Total pain score	Stage 1	Stage 2	Stage 3
Chi-square	8,312	,659	11,822	,997
df	2	2	2	2
Asymp. Sig.	,016	,719	,003	,607

Asymp. Sig.: asymptotic significance.

^a^Kruskal-Wallis test.

^b^Grouping variable: cataract type.

**(a):** 

Ranks			
	Age	*N*	Mean rank
Total pain score	40–59	17	23,62
	60–75	20	29,70
	76–97	19	31,61
	Total	**56**	

Stage 1	40–59	17	30,56
	60–75	20	28,50
	76–97	19	26,66
	Total	**56**	

Stage 2	40–59	17	21,32
	60–75	20	31,18
	76–97	19	32,11
	Total	**56**	

Stage 3	40–59	17	24,79
	60–75	20	30,00
	76–97	19	30,24
	Total	**56**	

*N*: number.

**(b):** 

Test statistics^a,b^				
	Total pain score	Stage 1	Stage 2	Stage 3
Chi-square	2,563	,912	5,763	2,199
df	2	2	2	2
Asymp. Sig.	,278	,634	,056	,333

Asymp. Sig.: asymptotic significance.

^a^Kruskal-Wallis test.

^b^Grouping variable: age.

**Table 7:** The relation between gender groups and both mean total pain score and mean pain score per stage.

	The mean pain score of group 1	The mean pain score of group 2	*P*
Total	**3.14**	**2.96**	0.956
Stage 1	0.77	0.72	0.643
Stage 2	1.25	1.27	0.817
Stage 3	1.11	0.96	0.248

Test: chi-square.

Group 1: males, group 2: females.

**Table 8:** The relation between laterality groups and both mean total pain score and mean pain score per stage.

	The mean pain score of group 1	The mean pain score of group 2	P
Total	**2.92**	**3.17**	0.194
Stage 1	0.70	0.79	0.440
Stage 2	1.29	1.24	0.084
Stage 3	0.92	1.13	0.175

Test: chi-square.

Group 1: right eyes, group 2: left eyes.
